# Supplementary material for: Soccer-related injuries utilization of U.S. emergency departments for concussions, intracranial injuries, and other-injuries in a national representative probability sample: Nationwide Emergency Department Sample, 2010 to 2013
Source: PLoS One. 2021 Oct 12;16(10):e0258345. doi: 10.1371/journal.pone.0258345 (PMC8509888; doi:10.1371/journal.pone.0258345)
Supplement: Attachment — Submitted filename: Response to Reviewers.pdf [file pone.0258345.s001.pdf]

Hans-Peter Simmen, MD

Academic Editor

PLOS ONE

Dear Dr. Simmen,

Thank you for this opportunity to revise and resubmit our article for reconsideration: "Soccer-related injuries utilization of U.S. emergency departments for concussions, intracranial injuries, and other-injuries in a national representative probability sample: Nationwide Emergency Department Sample, 2010 to 2013". You enclosed four requests from reviewers with our last correspondence. I have responded to each request.

1. We reviewed and formatted the manuscript according to the style requirements outlined by PLOS ONE.
2. The first and corresponding author is registered in the ORCID system and the ORCID ID is linked to the PLOS ONE profile of the primary and corresponding author.
3. We clarified that the study was classified as *not human research* by the UCLA South Campus Institutional Review Board. Specifically, our methods section describes the data as anonymous observations, devoid of any identifiers that could link these results to institutions or persons. In addition, we have abided by HCUP requirements that no data for 10 or fewer persons are reported in the analysis.
4. We reviewed the manuscript thoroughly and have included all figures and tables in the narrative, and included each following first reference to these data.
5. Based on reviewer comments, we also reran our analyses and included adjusted odds ratios derived from multivariable adjusted logistic regression analyses and included an additional figure.

Please note, this work is significant because we show the impact of soccer-related injuries presenting to ED is high, and remarkably costly, especially in those with an ICI diagnosis. We also believe this manuscript is appropriate to publish at PLOS ONE given the explicit aim to publish interdisciplinary research, and make it available to all, immediately, and without restriction.

Thank you for this opportunity to adjust our analyses and resubmit our finding for peer-review by PLOS ONE contributors. We look forward to your correspondence.

Sincerely,

Gerardo Flores, PhD, RN, MSN, MSK
gerardo.flores@ucla.edu
760-835-0515
